# Neurological assessment tool for screening infants during the first year after birth: The Brief‐Hammersmith Infant Neurological Examination

**DOI:** 10.1111/dmcn.15871

**Published:** 2024-01-29

**Authors:** Domenico M. Romeo, Chiara Velli, Francesca Sini, Elisa Pede, Graziamaria Cicala, Frances M. Cowan, Daniela Ricci, Claudia Brogna, Eugenio Mercuri

**Affiliations:** ^1^ Paediatric Neurology Unit Fondazione Policlinico Universitario A. Gemelli IRCCS Rome Italy; ^2^ Paediatric Neurology Unit Università Cattolica del Sacro Cuore Roma Rome Italy; ^3^ Department of Paediatrics Imperial College London UK; ^4^ National Centre of Services and Research for the Prevention of Blindness and Rehabilitation of Low Vision Patients—International Agency for the Prevention of Blindness (IAPB) Italia Onlus Rome Italy

## Abstract

**Aim:**

To develop a short version of the original Hammersmith Infant Neurological Examination (HINE) to be used as a screening tool (Brief‐HINE) and to establish if the short examination maintains good accuracy and predictive power for detecting infants with cerebral palsy (CP).

**Method:**

Eleven items were selected from the original HINE (‘visual response’; ‘trunk posture’; ‘movement quantity’; ‘movement quality’; ‘scarf sign’; ‘hip adductor angles’; ‘popliteal angle’; ‘pull to sit’; ‘lateral tilting’; ‘forward parachute reaction’; ‘tendon reflexes’) identifying those items previously found to be more predictive of CP in both low‐ and high‐risk infants. In order to establish the sensitivity of the new module, the selected items were applied to existing data, previously obtained using the full HINE at 3, 6, 9, and 12 months, in 228 infants with typical development at 2 years and in 82 infants who developed CP.

**Results:**

Brief‐HINE scores showed good sensitivity and specificity, at each age of assessment, for detecting infants with CP. At 3 months, a score of less than 22 was associated with CP with a sensitivity of 0.88 and a specificity of 0.92; at 6, 9, and 12 months, the cut‐off scores were less than 25 (sensitivity 0.93; specificity 0.87), less than 27 (sensitivity 0.95; specificity 0.81), and less than 27 (sensitivity 1; specificity 0.86) respectively. The presence of more than one warning sign, or items that are not optimal for the age of assessment, imply the need for a full examination reassessment.

**Interpretation:**

These findings support the validity of the Brief‐HINE as a routine screening method and the possibility of its use in clinical practice.

## Abstract 2

This original article is commented by Hewawitharana and Phillips on pages 1117–1118 of this issue.

This original article is linked to letter to the editor by Fehlings et al. on pages 1255–1257 of this issue.

**Abbreviations**
Brief‐HINE: Hammersmith Infant Neurological Examination, short version
HINE: Hammersmith Infant Neurological Examination

What this paper adds

- A short version of the Hammersmith Infant Neurological Examination (Brief‐HINE) has been provided as a routine screening method.
- Brief‐HINE scores showed good predictive accuracy from 3 to 12 months for detecting infants developing cerebral palsy.

Neurological examinations in infants are often used to detect neurological abnormalities and to predict motor function. One of the challenges of performing neurological examinations in infants is that some neurological responses change with increasing age in relation to the infant's maturation and development. Furthermore, in infants with brain lesions who are at risk of developing atypical neurological signs, the onset of such signs may often appear after several months from birth and a single assessment may not always correctly identify all children with having typical or atypical development.^1^ Ideally, each infant with even minimal risk factors should have comprehensive and sequential neurological examinations, but in practice this does not always happen because of limited resources and time.

The Hammersmith Infant Neurological Examination (HINE) has been extensively evaluated and found to be one of the most complete and, at the same time, simplest neurological examinations for the early detection of neuromotor impairment in both low‐ and high‐risk infants between 2 months and 24 months.^2^, ^3^, ^4^, ^5^, ^6^ Several papers have reported that the sequential use of the HINE is very helpful in the follow‐up of high‐risk infants during their first year, with available cut‐off scores at different ages for identifying early signs of cerebral palsy (CP).^3^ Although the HINE usually takes no longer than 15 minutes to complete, it is still considered too lengthy and too detailed by many paediatricians for use in routine clinical assessments.

To address these difficulties, we aimed to develop a screening assessment tool (Brief‐HINE) with a restricted number of HINE items that could be easily used in clinical practice, maintaining a similar power prediction as the full HINE in terms of early diagnosis and prognosis for CP.

## METHOD

### Development of the module

The original HINE, a neurological examination that is applicable for use in infants from 2 to 24 months, consists of 26 items including a number of items (e.g. trunk posture, lateral tilting, arm protection, and forward parachute) whose normal responses mature with the age and others that change little with age (e.g. visual and hearing responses, movement, arm posture, pull to sit, and abnormal signs).^2^, ^3^, ^4^, ^5^, ^6^

**FIGURE 1:**
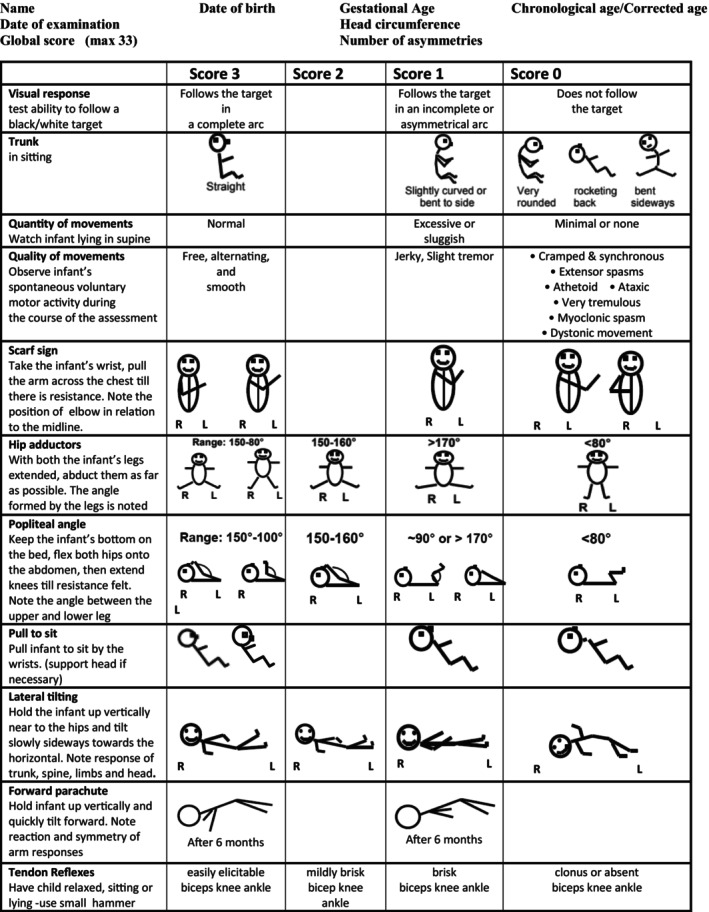
Brief‐Hammersmith Infant Neurological Examination (Brief‐HINE). Note: for trunk posture, lateral tilting, and forward parachute scores 0–1 are not considered as atypical at 3–6 months. For more information on how to perform each item see the HINE guidance notes that are available at: https://www.mackeith.co.uk/hammersmith‐neurological‐examinations/.

The selection of items for the new screening tool was based on our clinical and research experience, identifying the items from the HINE that had been previously found to be more predictive for CP in low‐ and high‐risk infants.^7^, ^8^, ^9^ These initially included 16 items: ‘visual response’; ‘trunk posture’; ‘foot posture’; ‘movement quantity’; ‘movement quality’; ‘scarf sign’; ‘pronation/supination’; ‘hip adductor angles’; ‘popliteal angle’; ‘pull to sit’; ‘ventral suspension’; ‘arm protection’; ‘vertical suspension’; ‘lateral tilting’; ‘forward parachute reaction’; ‘tendon reflexes’. Five of the 16 items were excluded as they were felt to be less easy to be reliably performed by clinicians with relatively little experience, especially in older infants (vertical suspension, ventral suspension, arm protection, foot posture, pronation/supination). The final selection totalled 11 items as shown on the Brief‐HINE proforma (Figure 1).

The Brief‐HINE maintains exactly the same format as the original HINE with no change in the performance or scoring of the items. The findings for each item can be documented on the form in one of four columns: column 1 (score 3) for findings frequently seen in the typically developing population (>75%) and considered optimal, column 2 (score 2) for findings less frequently found but still observed in more than 10% of the typically developing population, and columns 3 (score 1) and 4 (score 0) documenting findings seen in less than 10% of the population. Therefore the global Brief‐HINE scores can range from 0 to 33. The presence and the total number of asymmetries are also recorded. If an item has asymmetrical responses between left and right, the score is calculated as the average of the two scores if the asymmetries are in different columns (but the same score if they are in the same column).

In order to verify whether the new screening proforma correctly identifies infants with a normal outcome from those who developing CP, the module was applied retrospectively to 228 infants with typical development at 2 years and to 82 infants who developed CP. Both groups of infants included infants born at term, near‐term, and preterm routinely followed in our unit and previously assessed using the original full version of the HINE. This was possible as all the findings for the items contained in the screening proforma are included in the full examination.

All the infants described in this study were part of a follow‐up research project carried out at the Paediatric Neurology Unit of the Fondazione Policlinico Gemelli, Rome. All patients were enrolled routinely to a 2‐year follow‐up research protocol, which included HINE examinations at 3, 6, 9, and 12 months. The HINE was performed by two authors (DMR, DR) with an extensive experience in the field of paediatric neurology and in the administration and interpretation of the HINE with a good reliability as previously reported.^2^, ^3^, ^4^, ^5^, ^6^, ^7^, ^8^, ^9^ At the age of 24 months (corrected age if born preterm) they were routinely assessed using the neurological examination^10^ and the Griffith's Mental Development Scales, Second Edition.^11^ CP was identified according to Bax et al.'s definition.^12^

The study protocol was approved by the ethics committee of our institution (Fondazione Policlinico Gemelli) (ID 5326) and informed written consent was obtained from the parents in all cases.

### Statistical analysis

Brief‐HINE scores were reported as mean, standard deviation, and range at the four different ages, for the two cohort of infants (those with typical neurodevelopmental outcome and those with CP).

The measure of diagnostic accuracy of the Brief‐HINE global scores was determined by sensitivity and specificity of specific cut‐off scores, according to the age at assessment, for the presence of CP. Areas under the receiver operating characteristic curve were obtained and cut‐off values were estimated using the Liu method, which maximizes the product of the sensitivity and specificity.^13^ The level of significance was set at a *p*‐value less than 0.05. We used Stata statistical software version 15 (StatCorp, College Station, TX, USA) for the analysis.

## RESULTS

All the infants, including the 228 with typical 2‐year developmental outcome and the 82 with CP, completed all the items included in the original HINE (26 items) proforma at 3, 6, 9, and 12 months, and therefore could be rescored using the Brief‐HINE (11 items). Infants with typical developmental outcome included infants born at term (*n* = 65; 40 males, 25 females) and infants born preterm (*n* = 163; 98 males, 65 females). The 228 infants with typical neurodevelopmental outcome recorded at 2 years had a global quotient score of 85 or more on the Griffiths Scales and no CP. Of the 82 infants with CP (55 infants born preterm, 27 infants born at term), 25 were classified as having bilateral CP (quadriplegia) (15 males, 10 females), 17 as having spastic bilateral CP (diplegia) (10 males, 7 females), and 40 unilateral CP (23 males, 17 females).

### HINE item findings in the infants with typical neurodevelopmental outcome at 2 years (*n* = 228)

The mean global Brief‐HINE score in infants with a typical developmental outcome was 23 (SD 2.2; range 18–30) at 3 months with a gradual increase with increasing age reaching a mean of 31 (SD 1.6; range 27–33) at 12 months. Items with clinically detectable asymmetry (*n* = 1) were observed in 10 infants (0.4%). No infant had more than one asymmetry detected.

**FIGURE 2:**
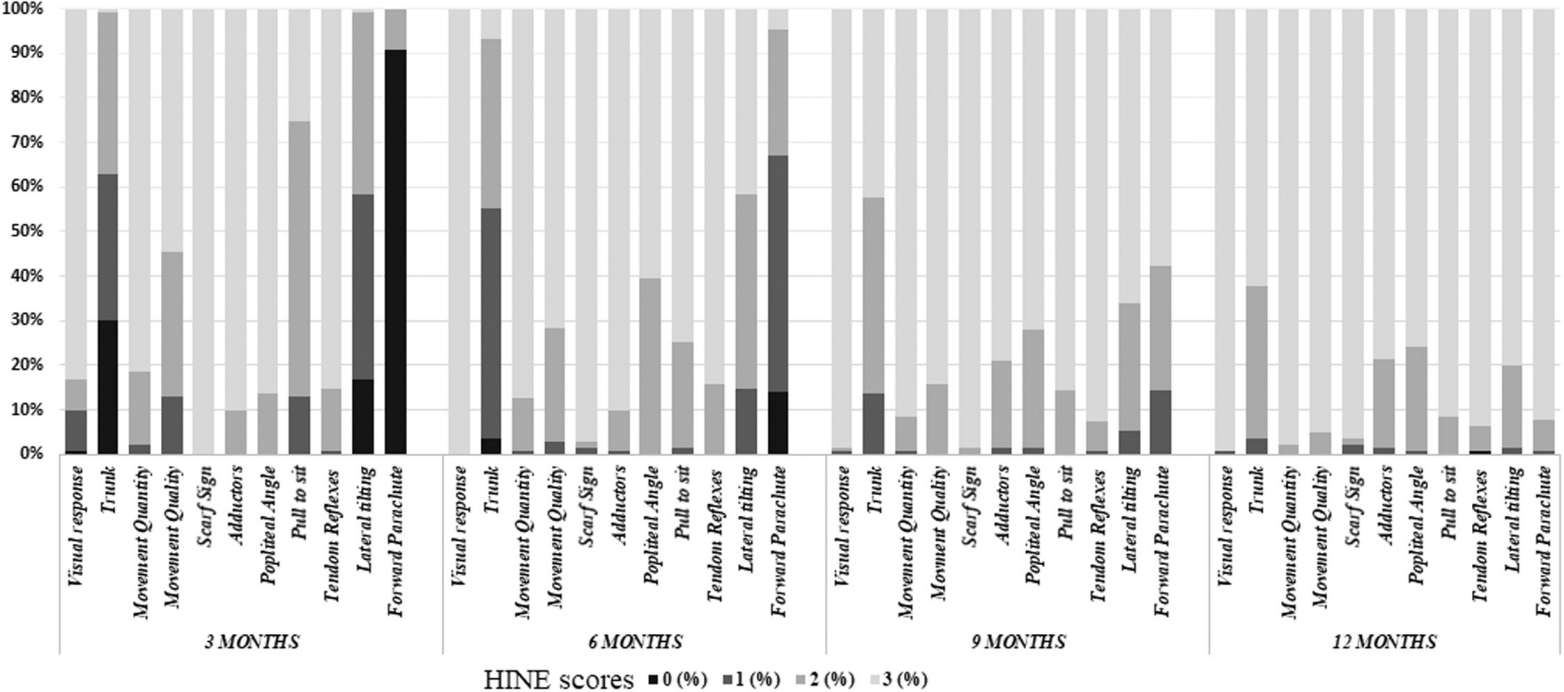
Distribution of Hammersmith Infant Neurological Examination item scores in the cohort of infants with a typical neurodevelopmental outcome at 2 years (*n* = 228).

Table 1 and Figure 2 shows the details of the single item and global scores of the Brief‐HINE as well as the scores achieved using the full HINE proforma.

**TABLE 1:** Distribution of the Hammersmith Infant Neurological Examination (HINE) and Brief‐HINE global scores in infants with typical neurodevelopmental outcome and those developing cerebral palsy and subtypes.

	Full HINE (max score 78) mean (SD; range)	Brief‐HINE (max score 33) mean (SD; range)
*3 months*		
Typical outcome	61 (4.8; 46–69)	23 (2.2; 18–30)
Cerebral palsy	44 (10; 14–58)	15 (4.9; 3–24)
Unilateral	55 (2.5; 51–58)	16.5 (10–24)
Bilateral (diplegia)	43 (6.5; 30–51)	14.5 (6–23)
Bilateral (quadriplegia)	30 (11.6; 14–39)	13.5 (3–18)
*6 months*		
Typical outcome	67 (3.4; 59–76)	28 (1.9; 22–32)
Cerebral palsy	49 (12.7; 16–66)	18 (5.8; 6–30)
Unilateral	59 (5.4; 54–66)	21 (13–30)
Bilateral (diplegia)	45 (4.7; 38–56)	20 (11–26)
Bilateral (quadriplegia)	29 (7.6; 16–36)	12.5 (6–21)
*9 months*		
Typical outcome	72 (3.7; 56–78)	30 (2.2; 20–33)
Cerebral palsy	49 (17.2; 16–67)	20 (7.3; 6–28)
Unilateral	62 (3.4; 57–68)	25.5 (18–28)
Bilateral (diplegia)	46 (8.9; 33–58)	22 (14–27)
Bilateral (quadriplegia)	27 (6.2; 16–31)	11 (6–21)
*12 months*		
Typical outcome	74 (3.2; 63–78)	31 (1.6; 27–33)
Cerebral palsy type	49 (21.1; 16–74)	22 (5.9; 10–33)
Unilateral	64 (5.1; 59–73)	25 (19–30)
Bilateral (diplegia)	49 (8.7; 38–61)	24.5 (21–33)
Bilateral (quadriplegia)	30 (11.2; 18–45)	12.5 (10–21)

### HINE item findings in the infants who were diagnosed with CP by 2 years (*n* = 82)

**FIGURE 3:**
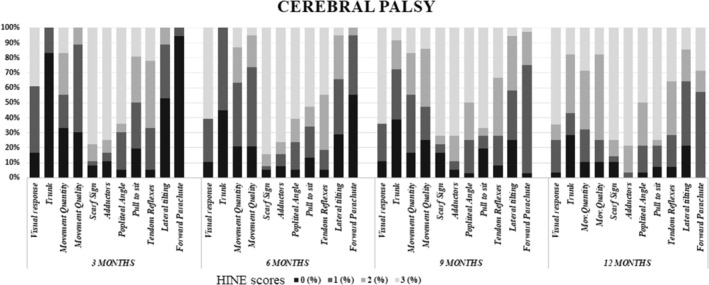
Distribution of Hammersmith Infant Neurological Examination item scores in the cohort of infants who developed cerebral palsy by 2 years (*n* = 82).

The mean global Brief‐HINE scores in infants who developed CP was 15 (SD 4.9; range 3–24) at 3 months with a gradual increase with increasing age. By 12 months, the mean score was 22 (SD 5.9; range 10–33). Table 1 and Figure 3 show details of the single item and global scores of the Brief‐HINE together with the scores achieved using the full HINE proforma.

**FIGURE 4:**
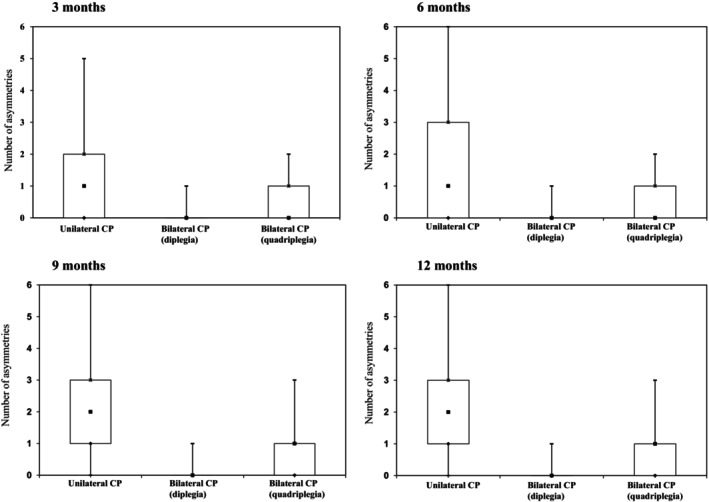
Number of asymmetries at different ages in infants with cerebral palsy (CP). Scatter plots show min–max (−) median (■) 25th centiles (♦) and 75th centiles (*).

Items with clinically detectable asymmetries ranged from 0 to 6 (median 1 at 3–6 months and median 2 at 9–12 months) in children developing unilateral CP, 0 to 2 in children developing bilateral CP (diplegia) (median 0, all ages), and 0 to 3 (median 0, all ages) in children developing bilateral CP (quadriplegia) (Figure 4).

### Number of ‘warning signs’

Scores of 0 and 1 fell always outside the 90th centile for 8 of the 11 items in the Brief‐HINE in the cohort of 228 infants with a typical neurodevelopmental outcome. These were ‘visual response’, ‘movement quantity’, ‘movement quality’, ‘scarf sign’, ‘hip adductor angles’, ‘popliteal angle’, ‘pull to sit’, and ‘tendon reflexes’. For the other three items (‘trunk posture’, ‘lateral tilting’, and ‘forward parachute’), scores were similar in both cohorts at 3 months and 6 months, as at this age a score of 0 or 1 is reported even in infants with typical neurodevelopmental outcome; scores of 0 and 1 were outside the 90th centile at 9 months and 12 months in the cohort with typical neurodevelopmental outcome. We therefore considered scores of 0 or 1 to be warning signs for atypical motor development for most items at all ages and for three items (‘trunk posture’, ‘lateral tilting’, and ‘forward parachute’) after 9 months and 12 months. For these three items, no warning signs should be considered at 3 months and 6 months.

**FIGURE 5:**
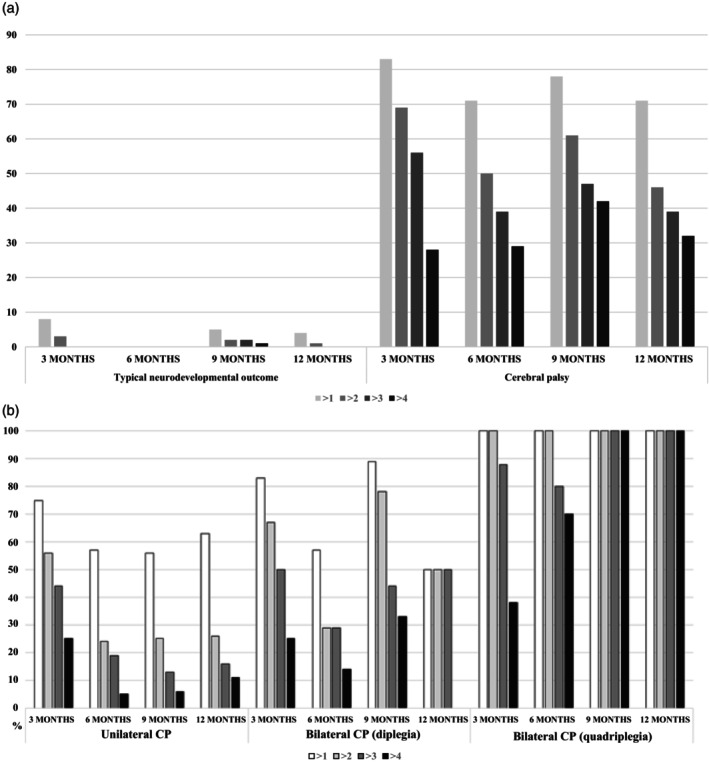
(a) Percentage of warning signs in infants with typical 2‐year outcome and those developing cerebral palsy at 3, 6, 9, and 12 months. (b) Percentage of warning signs in infants with different types of cerebral palsy.

For infants with a typical neurodevelopmental outcome (Figure 5a), at 3 months 8% had more than one warning sign and 3% had more than two warning signs, at 6 months no infant had more than one warning sign, at 9 months 5% had more than one warning sign, and at 12 months 4% had more than one warning sign.

For the cohort of infants who developed CP (Figure 5b), at 3 months 28% had more than four and 56% had more than three warning signs, at 6 months 39% had more than three warning signs and 50% had more than two warning signs, at 9 months and 12 months 70% to 80% had more than one and 30% to 40% had more than three warning signs. Details of the distribution of warning signs in the two populations are given in Figure 5.

### Cut‐off scores

At 3 months, a score of less than 22 was associated with CP with a sensitivity of 0.88 and a specificity of 0.92; at 6, 9, and 12 months, the cut‐off scores were less than 25 (sensitivity 0.93; specificity 0.87), less than 27 (sensitivity 0.95; specificity 0.81), and less than 27 (sensitivity 1; specificity 0.86) respectively. Table 2 shows details of the cut‐off.

**TABLE 2:** Cut‐off scores for cerebral palsy, according to the age at assessment.

Age at assessment (months)	Cut‐off	Sensitivity‐Specificity	PPV‐NPV‐ACC	AUC (95%CI)
3	22	0.88–0.92	0.8–0.95–0.91	0.90 (0.90–0.97)
6	25	0.93–0.87	0.72–0.97–0.88	0.91 (0.91–0.97)
9	27	0.95–0.81	0.65–0.98–0.85	0.88 (0.89–0.95)
12	27	1–0.86	0.72–1–0.9	0.94 (0.95–0.98)

Abbreviations: ACC, accuracy; AUC, area under the operating characteristic curve at cut‐off; CI, confidence interval; NPV, negative predictive value; PPV, positive predictive value.

## DISCUSSION

The HINE has been shown to be a reliable clinical neurological tool for the early identification of infants at risk of neurodevelopmental impairment.^2^, ^3^, ^4^, ^5^, ^6^, ^7^, ^8^, ^9^, ^14^, ^15^, ^16^ Because of time limitations in busy clinics and given that it can sometimes be difficult to examine young infants, it is not always easy to perform the full examination as a routine tool in paediatric follow‐up evaluations. In the present study, we have developed a new shorter version, the Brief‐HINE, to be used as a screening tool in low‐ and high‐risk infants.

The Brief‐HINE comprises a selection of items representing all sections of the original full HINE examination. These items were selected because they had been identified in previous studies and from our clinical experience to have better prediction for CP in low‐ and high‐risk infants.^7^, ^8^, ^9^ This short version of the HINE can be completed in less than 5 minutes and can be easily performed in a routine setting, allowing the examiner to keep a systematic record of these neurological items in a time‐efficient manner.

The brief version of the HINE includes a number of items, mainly related to trunk control and reflex reactions, that are age‐related in terms of their responses, allowing neurological maturation to be followed when the exam is used longitudinally. In infants with typical neurodevelopmental outcome, as previously reported using the full examination,^2^, ^3^, ^4^, ^5^, ^6^ a score of 3 in these items can be consistently found after 6 months only. In contrast, items such as those assessing limb tone or cranial nerves consistently have scores of 3 at 3 months of age onwards.

Despite the restricted number of items, asymmetries and early signs of unilateral CP could also be detected using the Brief‐HINE. When analysing the number of asymmetries on the Brief‐HINE in the group developing CP, we found that infants with unilateral CP had a higher number of asymmetries from 3 months of age. This has been previously reported by Hay et al. but was only statistically significant at a relatively late age (mean age of 15 months) when they used the asymmetry score from the full HINE to differentiate typically developing infants from those with unilateral CP.^16^

In the present study, we were also interested in establishing the diagnostic accuracy and predictive value of the Brief‐HINE to detect infants at risk of CP. Previous studies from our group and others have shown that the optimality score of the full HINE has a good predictive value for detecting infants who will develop CP.^2^, ^3^, ^4^, ^5^, ^6^, ^7^, ^8^ When we applied the same analysis to the results of the Brief‐HINE, we confirmed that the scores in infants with a typical outcome are different from those developing CP for both age‐related (‘trunk posture’, ‘lateral tilting’, and ‘forward parachute’) and non‐age‐related (‘visual response’, ‘movement quantity’, ‘movement quality’, ‘scarf sign’, ‘popliteal angle’, ‘pull to sit’, ‘hip adductors angle’, and ‘tendon reflexes’) items.

Our new findings confirm that, even when using the short version of the HINE, more than half of the infants who developed CP already have ‘atypical’ scores at 3 months. While some items, such as the ‘scarf sign,’ ‘adductors angle,’ ‘popliteal angle’, and ‘tendon reflexes’, are persistently atypical; others, such as ‘visual response,’ ‘movement quantity’, ‘movement quality’, and ‘pull to sit’, may show some improvement with increasing age, even if more than 30% of these items will still have an atypical score at 12 months.

Based on successful experience with the full HINE for identifying cut‐off scores predictive of atypical outcome, we applied the same method to the Brief‐HINE. The results showed that, even when using the short examination, it is possible to maintain similar high sensitivity and specificity for CP at any age of assessment.

These findings support the validity of the Brief‐HINE as a routine screening method, making it suitable for use in clinical practice. The good concordance and good prognostication do not suggest that the Brief‐HINE should completely replace the longer full HINE but rather be used as a screening method to identify infants who need a fuller neurological examination. We therefore suggest that whenever more than one warning sign is found (i.e. items that are not optimal for the age of assessment), a full examination should be performed as this will provide additional information that will be essential not only for confirming possible neurological abnormalities but also help in selecting further investigations.

The limitation of the present study concerns the retrospective application of the Brief‐HINE in infants developing CP with no sample size calculation. However, the number of infants included in the present study (*n* = 228) can be considered a sufficient and reliable number according to the literature about the HINE.^2^, ^3^, ^5^, ^7^, ^8^, ^9^ Further studies using the new proforma in prospective cohorts will help to further establish its value in identifying infants with other neurological conditions or with minor neurological impairments.

## Data Availability

Data available on request from the authors.
